# Carbohydrate-Based Macromolecular Crowding-Induced Stabilization of Proteins: Towards Understanding the Significance of the Size of the Crowder

**DOI:** 10.3390/biom9090477

**Published:** 2019-09-12

**Authors:** Sumra Shahid, Ikramul Hasan, Faizan Ahmad, Md. Imtaiyaz Hassan, Asimul Islam

**Affiliations:** 1 Centre for Interdisciplinary Research in Basic Sciences, Jamia Millia Islamia, Jamia Nagar, New Delhi 110025, India; 2 Department of Basic Medical Science, Faculty of applied Medical Sciences, Al-Baha University, PO Box: 1988- Al-Baha 65411, Saudi Arabia

**Keywords:** carbohydrate-based macromolecular crowder, protein folding, thermodynamic stability, crowder size, excluded volume

## Abstract

There are a large number of biomolecules that are accountable for the extremely crowded intracellular environment, which is totally different from the dilute solutions, i.e., the idealized conditions. Such crowded environment due to the presence of macromolecules of different sizes, shapes, and composition governs the level of crowding inside a cell. Thus, we investigated the effect of different sizes and shapes of crowders (ficoll 70, dextran 70, and dextran 40), which are polysaccharide in nature, on the thermodynamic stability, structure, and functional activity of two model proteins using UV-Vis spectroscopy and circular dichroism techniques. We observed that (a) the extent of stabilization of α-lactalbumin and lysozyme increases with the increasing concentration of the crowding agents due to the excluded volume effect and the small-sized and rod-shaped crowder, i.e., dextran 40 resulted in greater stabilization of both proteins than dextran 70 and ficoll 70; (b) structure of both the proteins remains unperturbed; and (c) enzymatic activity of lysozyme decreases with the increasing concentration of the crowder.

## 1. Introduction

The intracellular environment, where proteins fold and perform various functions, differs from the dilute buffer solutions often used during in vitro experiments. These dilute buffer solutions have been typically assumed to represent the in vivo scenario; however, there exists a major difference between the idealized (diluted) conditions and the environment present within cells [1,2,3]. The existence of a plethora of different macromolecules, including proteins, nucleic acids, ribosomes, and carbohydrates, makes the intracellular milieu extremely crowded by occupying around 10% to 40% of the total cellular volume [4]. It has been estimated that the overall concentration of macromolecules in the cytoplasm ranges from 80 to 400 mg mL^−1^ [1,3,5,6], restricting the space available to each individual molecule, and such a cellular condition has been termed as macromolecular crowding [7]. Macromolecular crowding indicates the presence of nonspecific steric repulsion between the molecules and generates the excluded volume effect, where any part of two macromolecules cannot exist in the same place at the same instant of time. In a cell, large macromolecules are present in such a way that a considerable portion of the intracellular space is unavailable to other molecules, hence reducing the accessible volume [8,9]. The presence of several macromolecules of different sizes, shapes, and compositions governs the level of crowding inside a cell [10]. Thus, the excluded volume effect should favor the folding reactions where the total volume, which is occupied by the molecules in the cytoplasm, is reduced. Moreover, theoretical models have predicted that macromolecular crowding should increase the stability of folded states of proteins due to unfavorable impacts on the unfolded state. A definite logical link between in vitro dilute conditions and in vivo crowded conditions is required to comprehend the process of protein folding and function in living organisms. Therefore, it is essential to determine how different degrees of macromolecular crowding alter the biophysical properties of proteins.

Since it is complicated to employ cell extracts in order to provide a natural microenvironment, different types of purified macromolecules are commonly used as synthetic crowding agents in experimental studies to scrutinize the macromolecular crowding effects [11,12]. It is widely accepted that a living cell is composed of macromolecular crowders in a variety of sizes and shapes [13]. However, it has been noticed that many studies have employed either fixed size crowders or in a narrow size range only to show their effects on proteins’ stability, structure and functional activity [8,11,14,15,16,17,18,19,20,21,22,23,24,25]. Moreover, many experiments have shown increase in the stability of proteins, although to different degrees [26,27,28,29,30,31,32,33,34,35,36,37,38,39,40], along with the alteration in the enzyme activity [41,42,43,44,45,46,47,48,49,50,51,52,53] in the presence of crowding due to the excluded volume effect. Thus, we found it necessary to examine the influence of crowding on different properties of proteins exerted by carbohydrate macromolecules of different sizes and shapes.

In this study, we investigated the effect of varying sizes of macromolecular crowders (ficoll 70, dextran 70, and dextran 40) on the thermodynamic stability of apo α-lactalbumin (α-LA) against thermal denaturation and its native and denatured structures. Similar experiments were carried out to see the effects of dextran 40 on the stability, structure, and functional activity of hen egg white lysozyme (some experiments in the presence of ficoll 70 and dextran 70 have already been done [54]). We evaluated ∆*G*_D_° (Gibbs free energy change at 25 °C) of α-LA and lysozyme, and kinetic parameters (*K*_m_, Michaelis constant; *k*_cat_, catalytic constant) of lysozyme in the absence and presence of crowders. Crowders of different molecular masses, shapes, and compositions were employed so as to meet the basic requirements of a cellular environment.

Osmolytes are small molecules accumulated by cells to protect them from denaturing stresses. These osmolytes protect cells from the hostile stresses by helping to maintain the structural and functional integrity of macromolecules. The crowders chosen in this study are polymers of the sugar osmolytes glucose and sucrose, which may have clinical implications for a large number of human diseases (e.g., amyloidosis, cancer, diabetes, and neurodegeneration) where intrinsically disordered proteins IDPs are involved [55]. Ficoll, a copolymer of sucrose and epichlorohydrin, is less flexible, highly branched, and compact (more like a sphere) [56,57,58]. Though, observations from dynamic light scattering (DLS) have shown that ficoll 70 assumes an intermediate shape between a sphere and a random coil [57]. Furthermore, ficoll was also modelled as a sphero-cylinder with a radius of about 14 Å [59]. However, dextran (polymer of D-glucose) is flexible, a linear polysaccharide with few and short branches, has a rod-like shape as well as a quasirandom coil molecule with a small number of short branches [56,57,58,60,61,62]. These crowding agents are highly soluble, inert, carbohydrate in nature, and do not intervene with spectroscopic experiments [8]. Their characteristic of being inert is crucial for associating theory and results since the excluded volume effect places an emphasis on steric repulsions [9,14].

In this study, we report that macromolecular crowding increases the stabilization of both α-LA and lysozyme, where the degree of stabilization is dependent on the shape and size of the crowding agents. The small size of dextran 40 excludes more volume and causes greater stabilization of proteins than ficoll 70 and dextran 70. The structural properties of both the proteins are unperturbed by macromolecular crowding. However, the functional activity of lysozyme is decreased with the increasing concentration of the crowding agent.

## 2. Materials and Methods

### 2.1. Materials and Reagents

Commercial lyophilized preparations of holo-α-lactalbumin from bovine milk, hen egg white lysozyme, ficoll 70 (F70; average molecular mass 70,000), dextran 40 and 70 (D40, D70; average molecular mass 40,000 and 70,000, respectively), sodium cacodylate trihydrate, and lyophilized cell wall of *Micrococcus luteus* were obtained from Sigma Aldrich Co. Potassium chloride, sodium acetate, glycine, sodium bicarbonate, ethylene diamine tetra acetic acid (EDTA), and ethylene glycol-bis (β-aminoethyl ether)-*N*,*N*,*N*’,*N*’-tetraacetic acid (EGTA) were purchased from Merck (India) Ltd. Guanidinium chloride (GdmCl) in ultrapure form was purchased from MP Biomedicals. All chemicals used were of analytical grade and used without further purification.

### 2.2. Preparation of Proteins and Reagents

Required amounts of holo-α-lactalbumin and lysozyme in lyophilized powdered form were dissolved in 0.1 M KCl solution at pH 7.0. We used the apo form of α-lactalbumin (α-LA) in our study, which was prepared by adding 5 mM EGTA to the solution of holo-α-lactalbumin (Ca^2+^ bound). Both the protein solutions were then dialyzed against several changes of 0.1 M KCl solution at pH 7.0 and 4 °C and filtered using a 0.22-µm Millipore syringe filter. The concentrations of lysozyme and α-LA solutions were determined experimentally using the molar absorption coefficient at 280 nm (*ε*_280_, M^−1^ cm^−1^) values of 39,000 and 29,210 for lysozyme [63] and α-LA [64], respectively. Protein stock solutions were stored at 4 °C. The stock solutions of GdmCl and macromolecular crowding agents (F70, D70, and D40) were prepared by dissolving their required amount in the desired buffer solutions and were degassed. All the solutions were then filtered through the Whatman filter paper No. 1 and the concentrations of GdmCl [65] and crowding agents [66,67] were determined by refractive index measurements.

All solutions used for optical measurements were prepared in the desired degassed buffers. For various pH/pH ranges, the buffers used were 0.1 M KCl-HCl buffer (pH 2.0), 0.05 M glycine-HCl (pH range 3.0–3.5), 0.05 M acetate buffer (pH range 4.0–5.0), and 0.05 M cacodylate buffer (pH range 6.0–7.0), all containing 0.1 M KCl. The pH of each solution was also measured after the activity and denaturation experiments in order to make sure that there was no change in pH during the experiments.

### 2.3. Thermal Denaturation Measurements

Thermal denaturation experiments of α-LA and lysozyme were carried out in a Jasco V-660 UV/Visible spectrophotometer equipped with a Peltier-type temperature controller (ETCS-761). The change in the absorbance of α-LA and lysozyme with an increasing temperature was followed at 295 and 300 nm, respectively. The concentrations of the proteins used were in the range 0.4–0.5 mg mL^−1^. Each sample was heated from 20 to 85 °C with a heating rate of 1 °C min^-1^ to provide adequate time for equilibration of the samples. All the measurements were performed in triplicate and approximately 650 data points of each transition curve were collected. Experiments were performed in the absence and presence of varying concentrations of F70, at different pH values; concentration of D70 and D40 was in the range 0–300 mg mL^−1^ and that of F70 in the range 0–350 mg mL^−1^. After denaturation, each protein sample was immediately cooled down to measure the reversibility of the reaction. All solution blanks were subtracted before analysis of the data. The raw absorbance data was converted into a change in the molar absorption coefficient (∆*ε*_λ_, M^−1^cm^−1^) at a given wavelength, λ. Each heat-induced transition curve was analyzed for *T*_m_ (midpoint of denaturation) and Δ*H*_m_ (enthalpy change at *T*_m_) using a non-linear least-squares analysis according to the relation:

(1) $$y\left(T\right)=\frac{y_{N}\left(T\right)+y_{D}\left(T\right)e^{\left[-\frac{\Delta H_{m}}{R}\left(\frac{1}{T}-\frac{1}{T_{m}}\right)\right]}}{1+e^{\left[-\frac{\Delta H_{m}}{R}\left(\frac{1}{T}-\frac{1}{T_{m}}\right)\right]}}$$

where *y*(*T*) is the optical property at temperature *T* (K), *y*_N_(*T*) and *y*_D_(*T*) are the optical properties of the native and denatured protein molecules at temperature *T* (K), and *R* is the gas constant. In the analysis of the transition curve, it was assumed that a parabolic function describes the dependence of the optical properties of the native and denatured protein molecules (i.e., *y*_N_(*T*) = *a*_N_ + *b*_N_*T* + *c*_N_*T*^2^ and *y*_D_(*T*) = *a*_D_ + *b*_D_*T* + *c*_D_*T*^2^, where *a*_N_, *b*_N_, *c*_N_, *a*_D_, *b*_D_, and *c*_D_ are temperature-independent coefficients) [68,69]. The value of the constant-pressure heat capacity change (Δ*C*_p_) was determined from the slope of the linear plots of Δ*H*_m_ versus *T*_m_ [70], i.e.,:

Δ*C*_p_ = (ძΔ*H*_m_/ძ*T*_m_)_p._ (2)

Using values of *T*_m_, Δ*H*_m_*,* and Δ*C*_p_*,* the value of Δ*G*_D_ at any temperature *T,* Δ*G*_D_(*T*) was estimated with the help of the Gibbs–Helmholtz equation:

(3) $$\Delta G_{D}\left(T\right)=\Delta H_{m}(\frac{T_{m}-T}{T_{m}})-\Delta C_{p}[(T_{m}-T)+T\ln(\frac{T}{T_{m}})]$$

### 2.4. Circular Dichroism (CD) Measurements

To characterize α-LA and lysozyme in terms of the secondary and tertiary structure, circular dichroism measurements were performed in a Jasco Spectropolarimeter (model J-1500) equipped with a circulation bath (MCB-100). Cells of a 0.1- and 1.0-cm path length were used for far-UV and near-UV CD, respectively. The far-UV and near-UV CD spectra of the native and denatured α-LA and lysozyme in the absence and presence of different concentrations of all the crowding agents (F70, D70, and D40) were recorded, and data acquisition was carried out using software provided by Jasco. At least three accumulations of the scanning were carried out for each sample to improve the signal to noise ratio, including the baseline, and all solution blanks were subtracted before the data analysis. N_2_ was flushed continuously through the machine at the rate of 5 lit min^-1^ and higher below 200 nm to minimize the noise level. Thermal denaturation of α-LA and lysozyme, in the absence and presence of the crowding agents (D70, D40, and F70), was monitored by following the changes in ellipticity, *θ*, at 222 nm in the temperature range of 20 to 85 °C with a heating rate of 1 °C. This heating rate provided adequate time for equilibration of the sample. The concentration used for both the proteins for far-UV CD measurement was 0.3 mg mL^−1^, however, for near-UV CD measurements, the concentration used was 0.4 mg mL^−1^ for α-LA and 0.4 mg mL^−1^ for lysozyme. The raw CD data (*θ*_λ_, *T*) were reduced to the concentration-independent parameter, the mean residue ellipticity, [*θ*]_λ_ (deg cm^2^ dmol^-1^), using the relation:

[*θ*]_λ_ =*M*_o_*θ*_λ_/10*lc,* (4)

where *θ*_λ_ is the observed ellipticity in millidegrees at wavelength, λ; *M*_o_ is the mean residue weight of the protein; *c* is the protein concentration in mg mL^−1^; and *l* is the path length of the cell in centimeters.

It should be noted that each experiment was performed in triplicate and the reversibility of thermal denaturation was determined under our experimental conditions by cooling the heated solution of denatured protein to 25 °C and comparing its optical property (either CD signal or absorbance) to that of the native protein before heating.

### 2.5. Activity Measurements

Following the classical turbidimetric method of Maurel and Douzou, the lytic activity of lysozyme with the *Micrococcus luteus* cell wall as a substrate was measured using a UV-Vis spectrophotometer (Jasco V-660) [71]. All activity measurements were performed in the presence of D40 as described in the procedure mentioned by Shahid et al. [54] in detail.

## 3. Results

### 3.1. Thermal Denaturation Study of α-LA

**Figure 1:**
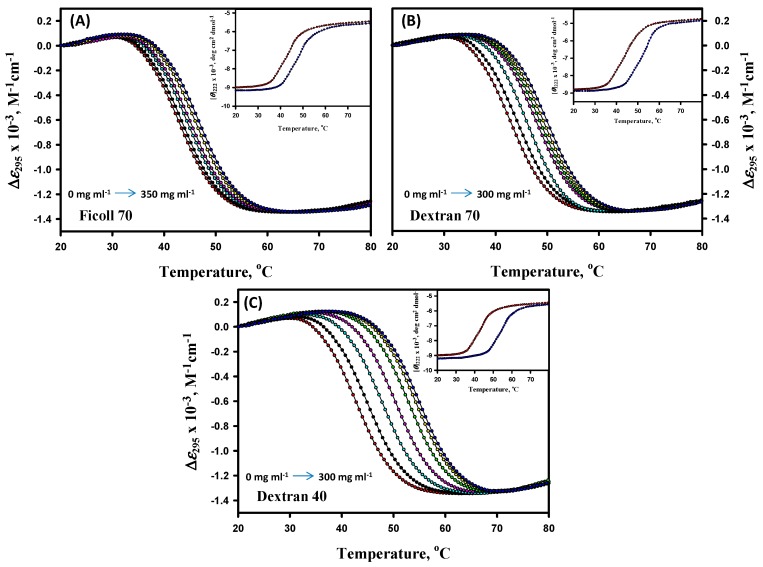
Thermal denaturation profiles of α-LA in the absence and presence of different concentrations of ficoll 70, dextran 70, and dextran 40 at pH 7.0 (panels **A**–**C**). Different concentrations of all the crowding agents are represented by different colors: 0 mg mL^−1^ (red), 50 mg mL^−1^ (black), 100 mg mL^−1^ (cyan), 150 mg mL^−1^ (pink), 200 mg mL^−1^ (green), 250 mg mL^−1^ (yellow) and 300 mg mL^−1^ (blue) (350 mg mL^−1^ in the case of ficoll 70). For the sake of clarity, curves at all concentrations are not shown. Insets in all the panels represent thermal denaturation profiles of α-LA measured by [*θ*]_222_ in the absence (red circle) and the presence of the highest concentration of the crowder (blue circle).

Thermal denaturation measurements of α-LA (pH values 7.0, 6.5, 6.0, and 5.5) were studied in the absence and presence of different concentrations of F70 (0–350 mg mL^−1^), D70 (0–300 mg mL^−1^), and D40 (0–300 mg mL^−1^) at the aforementioned pH values. Experiments were carried out by following changes in ∆*ε*_295_, the difference of the molar absorption coefficient (∆*ε*, M^−1^ cm^−1^) at 295 nm as a function of temperature. Since F70 rapidly hydrolyzes at pH values lower than 3.0, especially at elevated temperatures, experiments with F70 were carried out at pH values 7.0–4.0 [72]. Thermal denaturation was also monitored by [*θ*]_222_ in the presence of different concentrations of all the crowding agents at extreme pH values only. Figure 1 represents thermal denaturation profiles of α-LA in the absence and presence of different concentrations of F70, D70, and D40 at pH 7.0, however, thermal denaturation curves for other pH values are provided in the Supplementary Material (see Figures S1–S3). The insets of Figure 1 and Figures S1–S3 represent thermal denaturation profiles of α-LA monitored by following the changes in [*θ*]_222_. It is seen in these figures that the temperature dependencies of *y*_N_ and *y*_D_ measured by either ∆*ε*_295_ or [*θ*]_222_ do not depend on the concentration of the crowding agents at all the pH values. Thermal denaturation profiles of α-LA were found to be reversible in the absence and presence of the entire range of concentrations of all the crowding agents at all pH values (data not shown).

### 3.2. Analysis of Denaturation Curves of α-LA

Thermal denaturation curves of α-LA in the presence of different concentrations of a crowder were analyzed according to Equation (1) to obtain the values of *T*_m_ and Δ*H*_m_. Values of ∆*H*_m_ and *T*_m_ were obtained from three independent measurements. The average values of *T*_m_ and Δ*H*_m_ were plotted in the absence and presence of different concentrations of all the crowders at different pH values in order to estimate the value of the heat capacity change, Δ*C*_p_ from their slope using Equation (2). The reason for performing the experiments at different pH values was to estimate the Δ*C*_p_ of α-LA. The values of thermodynamic parameters (*T*_m_, Δ*H*_m_, and Δ*C*_p_) obtained in the absence and presence of varying concentrations of all the crowders at different pH values were then used to estimate the values of the Gibbs free energy change at 25 °C, Δ*G*_D_°, using Equation (3). The values of *T*_m_, ∆*H*_m_, ∆*C*_p_, and Δ*G*_D_° of α-LA in the absence and presence of varying concentrations of F70, D70, and D40 at pH 7.0 are given in Table 1, however, data for other pH values are provided in the Supplementary Material (see Tables S1–S3). Figure S4 show plots of ∆*H*_m_ versus *T*_m_ of α-LA in the absence and presence of different concentrations of F70, D70, and D40. It has been observed that with the increasing concentrations of all the three crowders, there was an increase in the values of Δ*G*_D_° of α-LA at all the pH values. These observations show that, on the mass (mg l^−1^) scale, an increase in *T*_m_ and Δ*G*_D_° was found to be more in the case of D40 when compared to D70 and F70 at pH 7.0 (a pH away from α-LA’s pI), demonstrating that D40 is stabilizing α-LA more than F70 and D70.

### 3.3. Thermal Denaturation Study of Lysozyme

**Figure 2:**
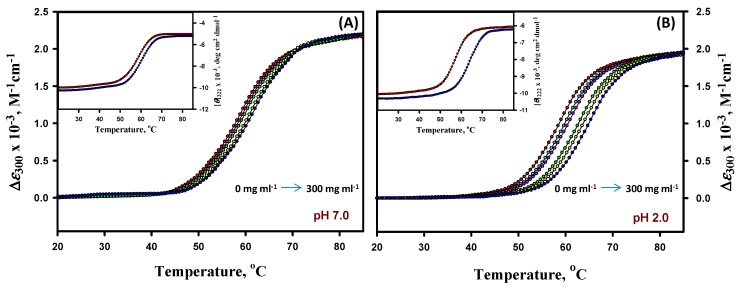
Thermal denaturation profiles of lysozyme in the absence and presence of different concentrations of dextran 40 at values pH 7.0 (panel **A**) and 2.0 (panel **B**). Different concentrations of dextran 40 are shown by different colors of circles, i.e., 0 mg mL^−1^ (red), 100 mg mL^−1^ (cyan), 150 mg mL^−1^ (pink), 200 mg mL^−1^ (green), 250 mg mL^−1^ (yellow), and 300 mg mL^−1^ (blue). For the sake of clarity, the curves at all concentrations are not shown. Insets in both the panels represent the thermal denaturation profiles of lysozyme measured by [*θ*]_222_ in the absence (red circle) and presence of the highest concentration of dextran 40 (blue circle).

Thermal denaturation measurements of lysozyme (pH values 7.0, 6.0, 5.0, 4.0, 3.0, and 2.0) were studied in the absence and presence of different concentrations of D40 (0–300 mg mL^−1^) at the abovementioned pH values. Experiments were carried out by following the changes in ∆*ε*_300_ (M^−1^ cm^−1^) as a function of the temperature. Thermal denaturation was also monitored by [*θ*]_222_ in the presence of different concentrations of all the crowding agents at extreme pH values only. Figure 2 represents the thermal denaturation profiles of lysozyme in the absence and presence of different concentrations of D40 at pH values 7.0 and 2.0; however, the thermal denaturation curves for other pH values are provided in the Supplementary Material (see Figure S5). The insets of Figure 2 represent thermal denaturation profiles of the lysozyme monitored by following the changes in [*θ*]_222_. It is seen in these figures that the temperature dependencies of *y*_N_ and *y*_D_ measured by either ∆*ε*_300_ or [*θ*]_222_ do not depend on the concentration of D40 at all the pH values. The thermal denaturation profiles were found to be reversible in the absence and presence of the entire range of concentrations of D40 at all pH values (data not shown).

### 3.4. Analysis of Denaturation Curves of Lysozyme

Thermal denaturation curves of lysozyme in the presence of different concentrations of D40 were analyzed according to Equation (1) to obtain the values of *T*_m_ and Δ*H*_m_. Values of ∆*H*_m_ and *T*_m_ were obtained from three independent measurements. The average values of *T*_m_ and Δ*H*_m_ were plotted in the absence and presence of different concentrations of D40 at different pH values in order to estimate the value of the heat capacity change, Δ*C*_p_ from their slope using Equation (2). The reason for performing the experiments at different pH values was to estimate the Δ*C*_p_ of lysozyme. The values of thermodynamic parameters (*T*_m_, Δ*H*_m_, and Δ*C*_p_) obtained in the absence and presence of varying concentrations of D40 at different pH values were then used to estimate the values of Gibbs free energy change at 25 °C, Δ*G*_D_° using Equation (3). The values of *T*_m_, ∆*H*_m_, ∆*C*_p_, and Δ*G*_D_° of lysozyme in the absence and presence of varying concentrations of D40 at pH 7.0 and 2.0 are given in Table 2, however, the data for other pH values are provided in the Supplementary Material (see Table S4). Figure S6 show plots of ∆*H*_m_ versus *T*_m_ of lysozyme in the absence and presence of different concentrations of D40. It was observed that with the increasing concentrations of D40, there was an increase in the values of Δ*G*_D_° of lysozyme at all the pH values. It was observed that on the mass (mg l^-1^) scale, there was an increase in *T*_m_ and Δ*G*_D_° of lysozyme with the increasing concentration of D40 at all the pH values, with the maximum increase and stabilization at pH 2.0 (the pH away from lysozyme’s pI).

**Figure 3:**
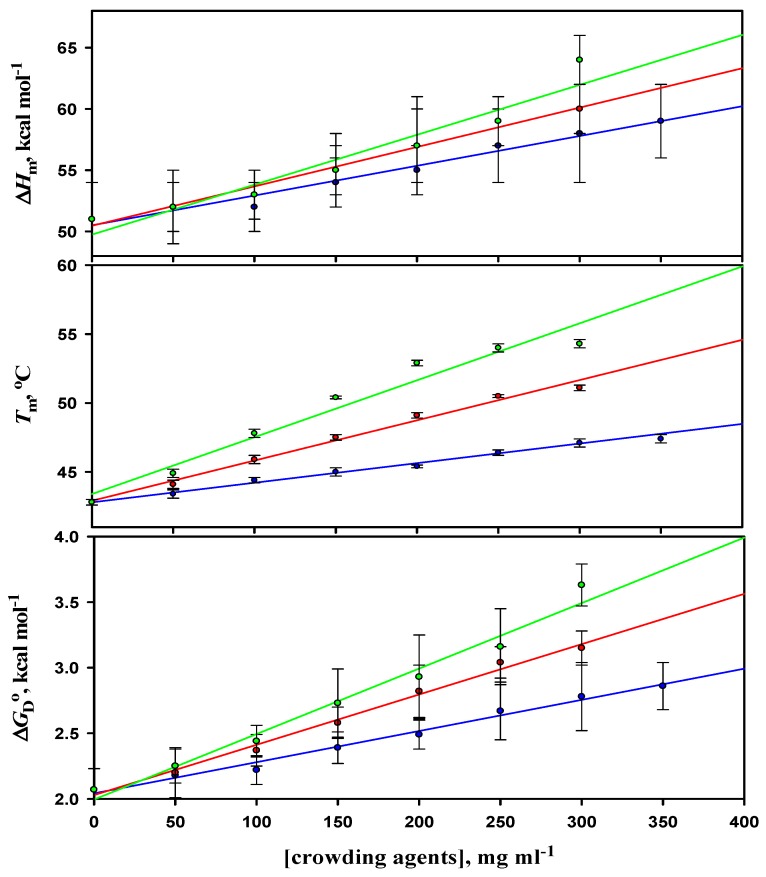
Plots of thermodynamic parameters (*T*_m_, Δ*H*_m_, and Δ*G*_D_°) associated with the thermal unfolding of α-LA versus the concentration of ficoll 70 (blue line), dextran 70 (red line), and dextran 40 (green line) at pH 7.0.

The thermodynamic parameters associated with the thermal unfolding of α-LA in the absence and presence of different concentrations of F70, D70, and D40 at pH value 7.0 are shown in Figure 3. The effects of all the three crowding agents on the thermodynamic stability of α-LA at different pH values are compared in Table 3.

**Figure 4:**
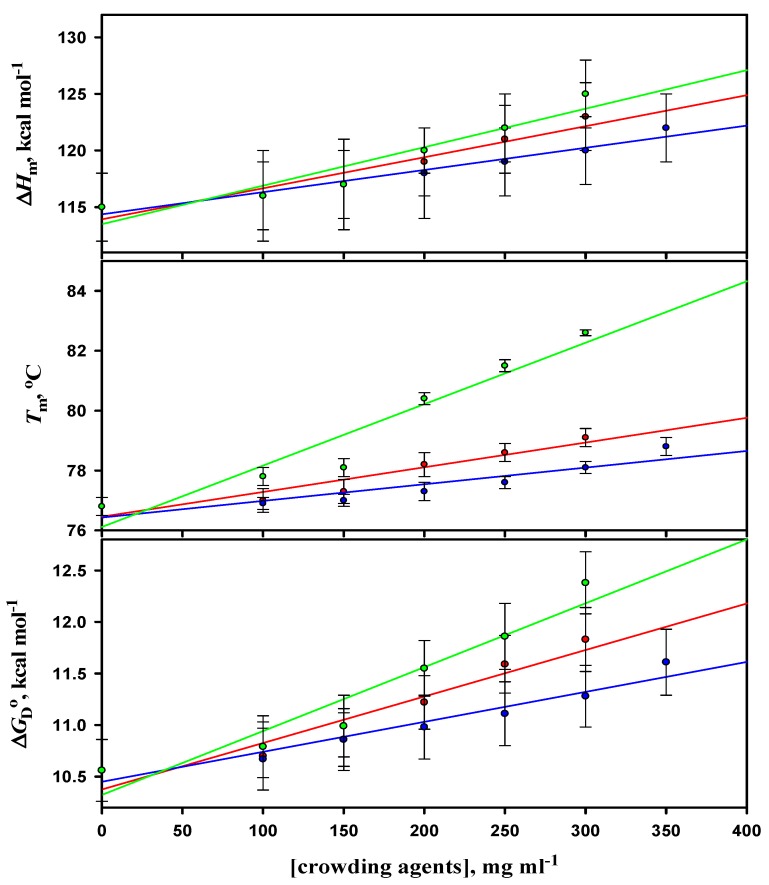
Plots of thermodynamic parameters (*T*_m_, Δ*H*_m_, and Δ*G*_D_°) associated with the thermal unfolding of lysozyme versus concentration of ficoll 70 (blue line), dextran 70 (red line), and dextran 40 (green line) at pH 4.0. The results for ficoll 70 and dextran 70 were taken from our previous published work.

The thermodynamic parameters associated with the thermal unfolding of lysozyme in the absence and presence of different concentrations of F70, D70, and D40 at pH value 4.0 are shown in Figure 4. The effects of all the three crowding agents on the thermodynamic stability of lysozyme at different pH values are compared in Table 4.

It should be noted that complete denaturation curves for lysozyme could not be obtained in the measurable temperature range at pH values 7.0 to 4.0. Thus, measurements were performed in the presence of 2.0 M GdmCl at these pH values so as to bring down the thermal denaturation in the measurable temperature range. Following a procedure published earlier [73], to correct values of thermodynamic parameters (*T*_m_ and ∆*H*_m_) for the effect of 2.0 M GdmCl, thermal denaturation measurements of lysozyme were carried out at other concentrations of the denaturant in the ranges 1.5–3.5 M at pH values 7.0 and 6.0 and 1.0–3.0 M at pH values 5.0 and 4.0 (denaturation curves not shown). Each transition curve measured at a given [GdmCl] and pH was analyzed to obtain the values of *T*_m_ and ∆*H*_m_ according to Equation (1). At a given pH, the values of *T*_m_ and ∆*H*_m_ were plotted against [GdmCl], which were found to be linear. Additionally, these linear plots were analyzed to measure the contribution of GdmCl to *T*_m_ and ∆*H*_m_. Intercepts of these linear plots were used to get the values of *T*_m_ and ∆*H*_m_ in the absence of GdmCl (data not shown).

### 3.5. Far- and Near-UV CD Measurements

**Figure 5:**
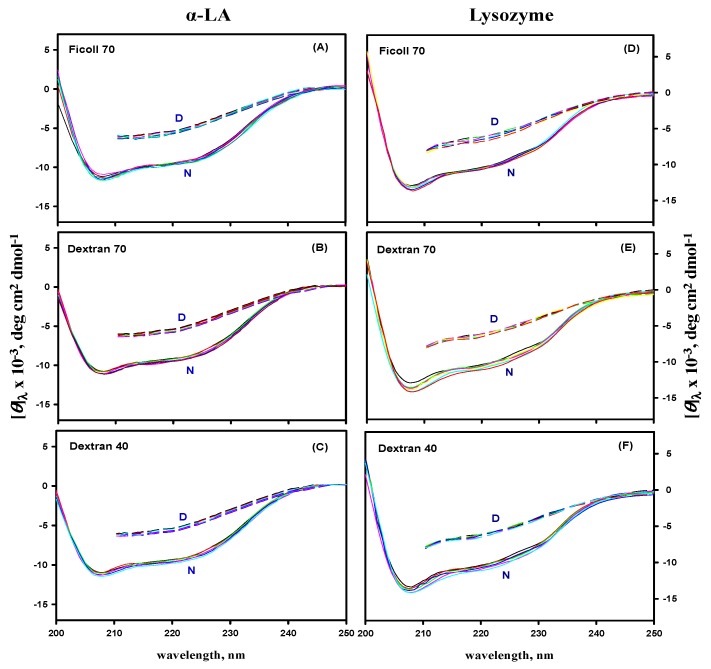
The far-UV CD spectra of the native and denatured states of α-LA (panels **A**–**C**) and lysozyme (panels **D**–**F**) in the absence and presence of different concentrations of ficoll 70, dextran 70, and dextran 40 at pH 7.0. N and D in each panel represent the native and denatured states of both the proteins, respectively.

**Figure 6:**
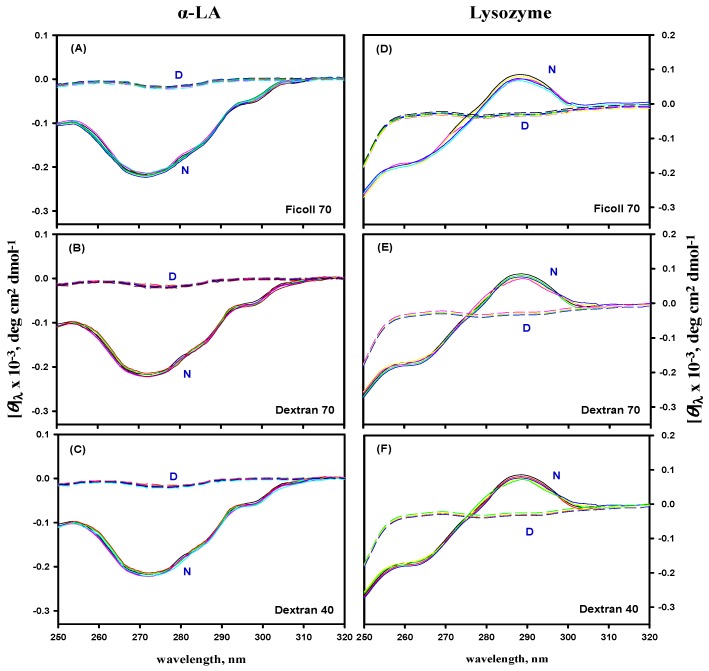
The near-UV CD spectra of the native and denatured states of α-LA (panels **A**–**C**) and lysozyme (panels **D**–**F**) in the absence and presence of different concentrations of ficoll 70, dextran 70, and dextran 40 at pH 7.0. N and D in each panel represent the native and denatured states of both the proteins, respectively.

The effect of macromolecular crowding on the secondary and tertiary structures of α-LA and lysozyme was investigated by recording their far- and near-UV CD spectra in the absence and presence of different concentrations of F70, D70, and D40 at pH 7.0. Figure 5 and Figure 6 show the far-UV and the near-UV CD spectra of the native (N) state at 25 °C, and denatured (D) state at 85 °C of α-LA and lysozyme in the absence and presence of different concentrations of all the crowding agents at pH 7.0, suggesting that there was no significant difference in the CD spectra of the N and D states of both the proteins.

### 3.6. Activity Measurements

**Figure 7:**
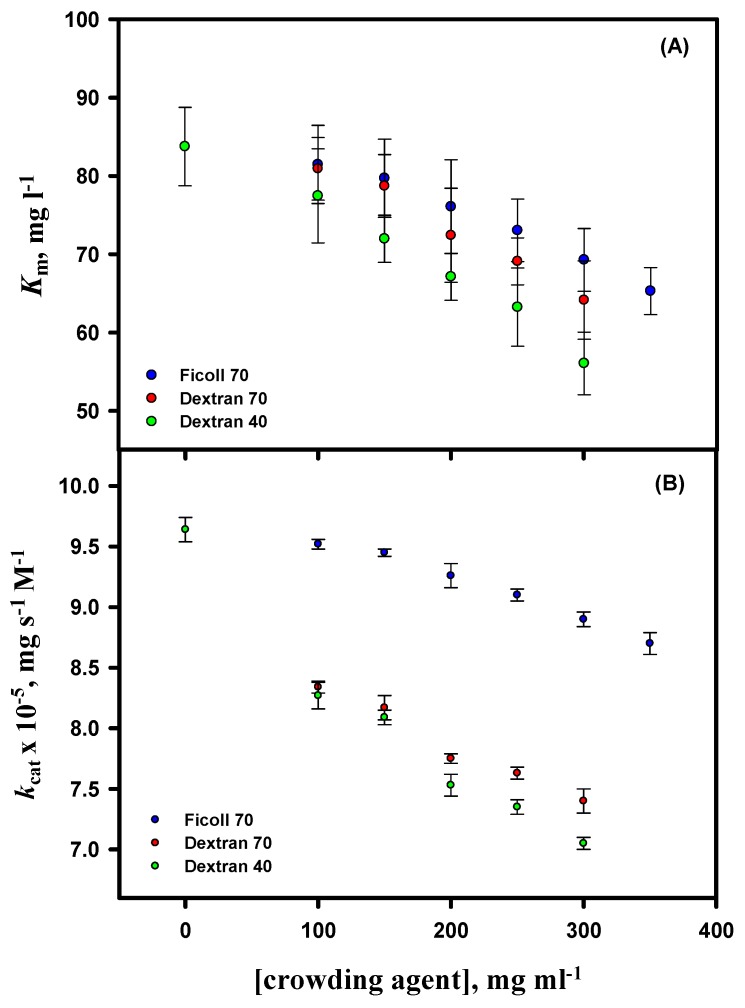
Plots of kinetic parameters *K*_m_ (panel **A**) and *k*_cat_ (panel **B**) of lysozyme versus the concentration of the crowding agent at pH 7.0 and 25 °C. Ficoll 70, dextran 70, and dextran 40 represent blue, red, and green circles, respectively. The results for ficoll 70 and dextran 70 are taken from our previous published work.

Functional activity measurements of lysozyme were carried out in the absence and presence of different concentrations of D40 at pH 7.0 and 25 °C (the activity measurements of lysozyme in the presence of F70 and D70 were already reported in our previous work [54]). For activity studies, the initial velocity (*v*) of the lysozyme-catalyzed reaction was determined at different substrate (*M. luteus*) concentrations. The Michaelis–Menten plots (parabolic curves) of lysozyme (*v* versus substrate concentration, [S]) in the absence and presence of D40 are provided in the Supplementary Material (Figure S7). A nonlinear least-square fit of the curves was used to determine the values of *K*_m_ and *V*_max_ of lysozyme in the absence and presence of different concentrations of D40 at pH 7.0 and 25 °C (see Equation (4) in [54]), and values of *k*_cat_ were estimated (see Equation (5) in [54]). Values of the kinetic parameters (*K*_m_ and *k*_cat_) of lysozyme in the absence and presence of D40 are provided in the Supplementary Material (see Table S5). The kinetic parameters in the absence and presence of highest concentration of D40 were: *K*_m_ = 83.75 ± 5.17 mg L^-1^ and 56.05 ± 4.07 mg L^-1^ and *k*_cat_ x 10^-5^ = 9.64 ± 0.09 mg s^-1^ M^-1^ and 7.05 ± 0.05 mg s^-1^ M^-1^, respectively. Values of *K*_m_ and *k*_cat_ of lysozyme were plotted against the concentration of each crowding agent (Figure 7). A decrease in the values of *K*_m_ and *k*_cat_ of lysozyme was observed with the increasing concentration of the crowding agents, and the effect was more pronounced for D40 than for F70 and D70 (the data can be compared from our previous work). The values of *K*_m_ and *k*_cat_ are the average of three independent measurements.

## 4. Discussion

The analysis of the thermal denaturation curves of α-LA and lysozyme in the absence and presence of different crowding agents was based upon two assumptions: (1) The transition between the native (N) and denatured (D) states of proteins is a two-state process; (2) crowding agents do not influence the structure of the N and D states of proteins. The measurements from differential scanning calorimetry (DSC) have demonstrated that the transition between the N and D states of both the proteins (α-LA and lysozyme) is indeed a reversible two-state process in the absence of crowders [70,74]. No such DSC measurements are reported for these proteins in the presence of F70, D70, and D40. To check the validity of the two-state assumption in the presence of crowders, we therefore measured thermal denaturation curves by following the changes in two different optical properties, namely ∆*ε*_λ_ and [*θ*]_222_, of α-LA and lysozyme in the presence of distinct concentrations of F70, D70, and D40 at different values. These transition curves, displayed in the insets of Figure 1, Figure 2, Figures S1–S3, and S5, were analyzed for *T*_m_ and ∆*H*_m_ and according to Equation (1), and these thermodynamic values were correlated with those attained from different absorption measurements (see Table 1, Table 2, and Tables S1–S3). It can be observed in Table 1, Table 2, and Tables S1–S3 that the thermodynamic parameters (*T*_m_ and ∆*H*_m_) of each protein in the presence of different concentrations of all three crowding agents are indistinguishable within the experimental errors. This observation is taken as an evidence for the two-state behavior of heat-induced denaturation of both proteins in the presence of crowding agents.

Since the thermodynamic parameters of α-LA and lysozyme reported in our study were attained by an indirect method based on equilibrium denaturation, it is essential that those values are validated against the values acquired directly by the calorimetric method. If ∆*H*_m_, *T*_m_, and ∆*C*_p_ of α-LA and lysozyme in the absence of crowders (see Table 1, Table 2, and Tables S1–S4) are compared with those collected from DSC measurements [70,74,75], then it is found that the values of each thermodynamic quantity obtained by these two distinct methods are in good agreement with each other. For example, the values of ∆*C*_p_ measured in our study are 1.56 ± 0.09 and 1.60 ± 0.09 kcal mol^-1^ K^-1^ for α-LA and lysozyme, respectively, and the calorimetric values of ∆*C*_p_ for the same are 1.55 and 1.60 kcal mol^-1^ K^-1^, respectively. Thus, it is believable that our measurements and analysis of transition curves for the thermodynamic parameters was done with accuracy and authenticity. Many studies have reported that thermal denaturation of several proteins, like hen-egg white lysozyme, bovine pancreatic RNase A, holo α-LA from bovine milk, horse heart cytochrome *c*, *P. aeruginosa* apoazurin, and monomeric Grx2, is a reversible two-state process in the presence of varying concentrations of different crowders at different pH values [27,34,35,37,40,76]. These findings are consistent with our reversible two-state unfolding of proteins in the presence of different crowding agents. Thus, based on several previous studies and our finding, it can be concluded that the thermal unfolding of both proteins is a reversible two-state process in the absence as well as the presence of different crowding agents at different pH values.

It has been noticed from the thermal denaturation curves of both the proteins that there is a shift in their transition region (see Figure 1 and Figures S1–S3 for α-LA and Figure 2 and Figure S5 for lysozyme) towards higher temperatures with increasing concentrations of each crowding agent (F70, D70, and D40), which signifies an increase in *T*_m_ (see Table 1 and Tables S1–S3 for α-LA and Table 2 and Table S4 for lysozyme) of proteins. An insignificant change was observed in the values of Δ*H*_m_ (within 10% experimental error) of both the proteins in the presence of all the crowders and at all the pH values. The purpose of our study was to scrutinize the effect of different concentrations of each crowding agent on Δ*G*_D_° (a key thermodynamic parameter signifying protein stability), estimated under all experimental conditions for both proteins (see Table 1 and Tables S1–S3 for α-LA and Table 2 and Table S4 for lysozyme). The values of Δ*G*_D_° for both the proteins increased with increasing concentration of all the crowders at all the pH values. This increase indicated the stabilization of proteins in the presence of crowding agents. Table 3 and Table 4 display the comparison of thermodynamic stability parameters of both the proteins in the presence of F70, D70, and D40 (300 mg mL^-1^) at different pH values. These tables consist of the values of change in *T*_m_, Δ*T*_m_ = (*T*_m (crowding agent)_ - *T*_m (buffer)_), and percent stabilization, %ΔΔ*G*_D_° = 100 × (Δ*G*_D_° _(crowding agent)_ - Δ*G*_D_° _(buffer)_ / Δ*G*_D_° _(buffer)_). As we move up from pH 5.5 to pH 7.0 in the case of α-LA and move down from pH 7.0 to pH 2.0 in the case of lysozyme (far from their pI values), the extent of stabilization increases along with the increase in the concentration of crowders. In other words, there is a decrease in the stability of α-LA and lysozyme as the pH progressively deviates from their pI values, i.e., ~4.5 and ~11, respectively [77]. Nevertheless, it is perhaps qualitatively correct to state that as the pH is reduced from 7.0 to 5.0, the net charge on α-LA reduces; however, the net charge on lysozyme increases when the pH decreases from 7.0 to 2.0 [77]. Thus, the electrostatic hypothesis by Beg et al. [77] predicted that with the decrease in pH, the stabilizing influence of all sugars upon α-LA as quantified by the degree of *α* (a temperature-independent function of the sizes and shapes of the protein and sugar) will reduce, but in the case of lysozyme it will increase. It was also predicted that the conformation of the D state will turn out to be even more extended with the rising net charge [77]. Thus, it was noticed that the degree of stabilization is pH dependent, which is consistent with the observations reported earlier [37,40,77,78,79]. In addition, the order of stabilization in terms of the crowder’s size and shape was found as follows: Dextran 40 > Dextran 70 > Ficoll 70 for both the proteins. The calculated values of Δ*T*_m_ and %ΔΔ*G*_D_° suggested that maximum stabilization of α-LA and lysozyme was attained in the presence of the highest concentration of D40. D40 was found to be the most stabilizing crowding agent among the three crowders. The percent stabilization of α-LA is highest in the presence of 300 mg mL^-1^ of D40, i.e., 75.36% followed by D70, i.e., 52.17% and then F70, i.e., 34.29% at pH 7.0 (see Table 3). Plots of *T*_m_, Δ*H*_m_, and Δ*G*_D_° against different concentrations of all the three crowders for α-LA are shown at pH 7.0, and the results were compared (see Figure 3). Similarly, the percent stabilization of lysozyme is maximum for 300 mg mL^-1^ D40 (i.e., 33.51%) followed by D70 (i.e., 25.84%) at pH 2.0 and then by F70 (i.e., 6.81%) at pH 4.0 (see Table 4). Since we were unable to conduct the experiments below pH 4.0 in the case of F70 in our previous work [54], plots of *T*_m_, Δ*H*_m_, and Δ*G*_D_° against different concentrations of all three crowders for lysozyme are shown at pH 4.0 only for comparing the data (see Figure 4). It is assumed that the low average molecular mass of D40 in comparison to D70 and F70 could be the reason behind its ability to stabilize both the proteins more. On the scale of mass/volume, D40 has a greater number of molecules than D70, which further leads to maximum packing in the system and gives rise to the highest excluded volume. Thus, the small size of D40 causes maximum volume exclusion and hence stabilization of both the proteins more than D70 and F70. As far as shape is concerned, the molecules of dextran have shown greater stabilization of both the proteins than the molecules of ficoll. Ficoll is rigid, compact, and different in shape than dextran, exhibiting a compressed structure and consequently a smaller excluded volume effect than expected [45]. Thus, dextrans might have brought a greater effect on the stability of both the proteins with the ability of excluding more volume as compared to ficoll. The shape of a crowding agent has been supposed to play an essential role in bringing about the changes in excluded volume effects and hence the stabilization of proteins. Some of the properties of dextran, while in solution, are associated with the degree of branching for instance intrinsic viscosity, Stokes radius, and radius of gyration; however, these parameters do not illustrate the type, extent, length, or even the branches’ distribution [80]. A more linear structure is supposed to be found for low molecular mass, since the structure of dextran changes with the molecular size, whereas a structure with more branches can be discovered for a higher molecular mass [81]. Additionally, some studies have controverted the behavior of ficoll as an idealized compact spherical molecule [57,82]. It has been debated that ficoll, when dissolved in body fluids, converts from a hard sphere to a rather deformable molecule as a result of interactions between Na^+^ ions and weakly charged groups (some of the numerous end groups existing in a highly branched and cross-linked ficoll might have gone through oxidation to form carboxylic acid residues) within the molecule. Subsequently, it has been demonstrated that the ficoll molecule can adsorb Na^+^ ions (or K^+^ ions), due to which it takes on the properties of a pseudopolycation [83,84]. Moreover, Wenner et al. [85] proposed that ficoll acts as an open and deformable structure in crowded solutions. Thus, instead of strictly believing ficoll to be a compact sphere, it can also be considered as a slightly deformable sphere.

Radical contributions have been made by Minton in elucidating the significance of crowding in biophysics as he was the first to identify that the thermodynamics of biochemical reactions, association, and folding can be modified by crowding agents [7,11,14,86,87,88]. The non-specific interactions, considered as background interactions by Minton, are the interactions between the macromolecules and their proximate surroundings [89]. These background interactions among the molecules are dependent on their properties like size, shape, and concentration. A system or a cell is said to be crowded when a macromolecule is enclosed by other macromolecules occupying a considerable portion of the available volume [89]. These background interactions substantially decrease the accessible volume in the cell and consequently do not play any role directly in the reaction. Thus, the influence of macromolecular crowding on the stability of protein is best elucidated by the excluded volume effect. The excluded volume to the center of mass of a macromolecule is greatly influenced by the surface to volume ratio, and if the occupied volume by macromolecules is equivalent, then the surface area of macromolecules plays an indispensable part in determining the stability of proteins [2,90]. This volume exclusion phenomenon has been indicated to be the chief performer in stabilizing proteins under a crowded milieu [91,92,93,94,95]. The most fundamental attribute of the excluded volume effect is that all the solute molecules are mutually impenetrable in a system [9,14]. Nonspecific steric repulsion always exists between molecules irrespective of any other repulsive or attractive interactions [9]. Hence, the preferential exclusion of the crowding agents from the protein domain owing to macromolecular crowding leads to stabilization of the native state by transferring the equilibrium, N ↔ D, towards the N state. Thus, when a crowder is present, there is a transformation of denatured molecules into the native molecules since it leads to an increase in Gibbs free energy change related with the denaturation equilibrium [7,96]. There are several studies that provide evidences for the stabilizing nature of macromolecular crowding agents. These studies [26,27,28,29,30,31,32,33,34,35,36,37,38,39,40,54,76,97,98,99,100,101,102,103,104,105,106,107,108,109,110,111,112,113,114,115] have shown that macromolecular crowding enhances the thermodynamic stability of proteins. Any reaction bringing about a change in volume is affected by macromolecular crowding owing to excluded volume effects [116,117]. It has been suggested by Zhou and Cheung et al. [28,118] that crowding provides a stabilizing effect indirectly to the folded states of proteins as a result of the destabilization of the extended and unfolded states. It was also observed that crowding agents raise the thermodynamic stability of both the proteins without causing much change to their enthalpy of unfolding or we can suggest that crowding-induced stabilization of proteins is entropic in nature. The entropically driven stabilization of the folded state in the presence of the crowding environment [27,28,86,119] has been firmly recognized in several theoretical studies. It has been found that the values of ∆*C*_p_ of both the proteins are independent of the concentrations of the crowders, causing no change in ∆*C*_p_ upon transporting it from water to the solution of crowders (see Table 1, Tables S1–S3 for α-LA and Table 2 and Table S4 for lysozyme). It is assumed that the ∆*C*_p_ of a protein is associated to the alterations in the polar as well as the nonpolar accessible surface areas when protein denaturation takes place [120]. Thus, no change in ∆*C*_p_ denotes that polar and nonpolar involvements of ∆*C*_p_ stay either unaltered or altered in such a way that they bring about no modification in the monitored heat capacity change of both the proteins [54].

Furthermore, in order to validate the second assumption, we monitored the far- and near-UV CD spectra of the N and D state of both the proteins in the absence and presence of different crowding agents (see Figure 5 and Figure 6). It was observed that crowding agents do not affect the native as well as denatured states of secondary and tertiary structures of α-LA and lysozyme. Several studies have been reported, revealing that crowding conditions do not cause any significant perturbations in the native [37,40,47,121,122] and denatured states [37] of the structure of proteins, supporting our assumption. However, a few studies have also reported that some of the crowding agents cause changes in the structure of proteins [29,30,46,103]. Thus, this led to an assumption that changes in the structural contents of proteins in the macromolecular crowding environment can be protein specific.

In our previous study [54], we demonstrated the effect of different concentrations of two crowding agents of different shapes but similar sizes (F70 and D70) on the functional activity of lysozyme, showing that the excluded volume effect plays a substantial role in altering the functional activity of the protein. Thus, we further carried out the same experiments in the presence of D40 in order to examine the consequence of the different sizes of a crowder on the kinetic parameters (*K*_m_ and *k*_cat_) of lysozyme. It was observed that the values of *K*_m_ and *k*_cat_ of lysozyme decrease with the increasing concentration of each crowding agent [54], but the effect is more noticeable in the case of D40 followed by D70 and then F70. The crowders may have a larger influence on the encounter rates or diffusion of the enzyme and substrate owing to the volume exclusion and a rise in the solution viscosity that will eventually lead to reduced rates of molecular diffusion and hence enzymatic activity [41,90]. As explained in our previous work [54], the decrease in *K*_m_ of lysozyme could be an increment in the chemical activity of small substrate molecules in a highly non-ideal crowded condition, or when there is a rise in the ratio of activity coefficients between the enzyme and the enzyme–substrate complex [41,53,101,123,124]. Additionally, the crowding-induced alterations of the surroundings of an enzyme lead to conformational changes of an enzyme active site, which eventually results in a reduction in the values of *V*_max_, thereby affecting the values of *k*_cat_ [12,50,90,101,123,124,125]. Our results are in accord with most of the studies done on the effect of macromolecular crowding on enzymatic reactions [12,41,43,85,90,101,123,124,126,127]. Although crowding led to a decrease in the kinetic parameters of lysozyme, D40, among the three crowders, due to its low average molecular mass and hence a large number of molecules (on the mass/volume scale), this lead to the highest packing and a greater excluded volume effect. In addition, the behavior of *k*_cat_ dependency and *K*_m_ independency on the type of crowder could be credited to the dissimilarities in their shapes [54] but not size (see Figure 7). Moreover, the discrepancy in the conduct of *k*_cat_ in the presence of dextrans in comparison to ficoll with the increase in their concentrations can also be accredited to the disparities in their viscosities. The greater viscosity of dextrans [128] than ficoll [129,130,131] can possibly explain the behavior of *k*_cat_. Thus, based on the consequences achieved from our previous [54] and present experiments, we can assume that both the shape and size of a crowder play a vital role in altering the functional activity of a protein. In other words, the enzymatic reaction rate is significantly influenced by the contribution of the architecture of the crowder.

## 5. Conclusions

The study revealed that macromolecular crowding stabilizes α-LA and lysozyme, and D40 is a better protein stabilizer than F70 and D70. On the mass/volume scale, D40 has more molecules than D70, which results in maximum packing and hence the highest excluded volume. The crowding-induced stabilization of proteins is entropic in nature and displays pH-dependence. Moreover, the functional activity of lysozyme declines with the rising concentration of the crowding agents. Hence, the size and shape of the crowders were found to play a substantial role in influencing the biophysical properties of a protein in an intracellular environment. Thus, in order to attain a generalized interpretation of the biological processes in vivo, it is essential that the biological properties and processes in the presence of different degrees of crowdedness are examined. The effect of these different levels of crowdedness on protein stability and proteins’ biophysical properties can vary considerably with time and such variation may play a significant role in protein aggregation-related diseases, like Parkinson’s and Alzheimer’s disease. Since different osmolytes have functional and structural consequences on IDPs, they may assist in enhancing the disease pathology of various human diseases (such as amyloidosis, neurodegeneration, cancer, and diabetes), with the aggregation of IDPs as the common hallmark [55]. Hence, the crowders (polymers of sugar osmolytes) used in our study may have clinical implications for diseases including IDPs.

## Figures and Tables

**Table 1:** Thermodynamic parameters associated with the thermal denaturation of α-LA in the absence and presence of varying concentrations of ficoll 70, dextran 70, and dextran 40 at pH 7.0 **^a,b^**.

[Crowder] (mg mL^−1^)	*T*_m_ (^o^C)	∆*H*_m_ (kcal mol^−1^)	Δ*C*_p_ (kcal mol^−1^ K^−1^)	∆*G*_D_^o^ (kcal mol^−1^)
**0**	42.8 ± 0.2 (42.9 ± 0.2)	51 ± 3 (51 ± 3)	1.56 ± 0.09	2.07 ± 0.16 (2.08 ± 0.15)
**Ficoll 70**				
**50**	43.4 ± 0.3	52 ± 3	1.55 ± 0.09	2.18 ± 0.21
**100**	44.4 ± 0.2	52 ± 2	1.58 ± 0.06	2.22 ± 0.11
**150**	45.0 ± 0.3	54 ± 2	1.57 ± 0.08	2.39 ± 0.12
**200**	45.4 ± 0.1	55 ± 2	1.55 ± 0.07	2.49 ± 0.11
**250**	46.4 ± 0.2	57 ± 3	1.56 ± 0.07	2.67 ± 0.22
**300**	47.1 ± 0.3	58 ± 4	1.57 ± 0.06	2.78 ± 0.26
**350**	47.4 ± 0.3 (47.5 ± 0.2)	59 ± 3 (59 ± 2)	1.57 ± 0.09	2.86 ± 0.18 (2.87 ± 0.15)
**Dextran 70**				
**50**	44.1 ± 0.2	52 ± 3	1.58 ± 0.09	2.20 ± 0.19
**100**	45.9 ± 0.3	53 ± 2	1.57 ± 0.07	2.37 ± 0.12
**150**	47.5 ± 0.2	55 ± 2	1.58 ± 0.07	2.58 ± 0.12
**200**	49.1 ± 0.2	57 ± 3	1.56 ± 0.08	2.82 ± 0.20
**250**	50.5 ± 0.1	59 ± 2	1.55 ± 0.06	3.04 ± 0.12
**300**	51.1 ± 0.2 (51.0 ± 0.2)	60 ± 2 (60 ± 3)	1.55 ± 0.09	3.15 ± 0.41 (3.15 ± 0.16)
**Dextran 40**				
**50**	44.9 ± 0.3	52 ± 2	1.57 ± 0.09	2.25 ± 0.13
**100**	47.8 ± 0.3	53 ± 2	1.59 ± 0.07	2.44 ± 0.12
**150**	50.4 ± 0.1	55 ± 3	1.55 ± 0.08	2.73 ± 0.26
**200**	52.9 ± 0.2	57 ± 4	1.58 ± 0.08	2.93 ± 0.32
**250**	54.0 ± 0.3	59 ± 3	1.56 ± 0.09	3.16 ± 0.29
**300**	54.3 ± 0.3 (54.3 ± 0.2)	64 ± 2 (64 ± 2)	1.55 ± 0.09	3.63 ± 0.16 (3.63 ± 0.16)

**^a^** A ‘±’ sign with each parameter represents the mean error obtained from triplicate measurements. **^b^** Each value in parenthesis is measured by [*θ*]_222_.

**Table 2:** Thermodynamic parameters associated with the thermal denaturation of lysozyme in the absence and presence of varying concentrations of dextran 40 at pH 7.0 and 2.0 **^a,b^**.

[Dextran 40] (mg mL^−1^)	*T*_m (obs.)_^c^ (^o^C)	*T*_m (corr.)_^d^ (^o^C)	∆*H*_m (obs.)_^c^ (kcal mol^−1^)	∆*H*_m (corr.)_^d^ (kcal mol^−1^)	Δ*G*_D_º (kcal mol^−1^)
**pH 7.0**					
**0**	59.9 ± 0.3 (59.8 ± 0.3)	85.5 ± 0.3 (85.4 ± 0.3)	93 ± 3 (93 ± 4)	127 ± 3 (127 ± 4)	12.80 ± 0.30 (12.75 ± 0.27)
**100**	60.1 ± 0.3	85.7 ± 0.3	94 ± 3	128 ± 3	12.98 ± 0.40
**150**	60.4 ± 0.2	86.0 ± 0.2	95 ± 2	129 ± 2	13.21 ± 0.26
**200**	60.8 ± 0.2	86.4 ± 0.2	97 ± 3	131 ± 3	13.57 ± 0.24
**250**	61.7 ± 0.2	87.3 ± 0.2	99 ± 4	133 ± 4	13.88 ± 0.40
**300**	62.0 ± 0.3 (61.9 ± 0.3)	87.6 ± 0.3 (87.5 ± 0.3)	100 ± 2 (99 ± 2)	134 ± 2 (133 ± 2)	14.06 ± 0.34 (13.89 ± 0.31)
**pH 2.0**					
**0**	57.6 ± 0.2 (57.6 ± 0.2)	-	84 ± 2 (84 ± 2)	-	5.61 ± 0.13 (5.61 ± 0.13)
**100**	58.8 ± 0.2	-	86 ± 2	-	5.92 ± 0.19
**150**	59.5 ± 0.3	-	88 ± 2	-	6.19 ± 0.23
**200**	61.8 ± 0.2	-	92 ± 3	-	6.78 ± 0.28
**250**	62.9 ± 0.3	-	94 ± 3	-	7.06 ± 0.29
**300**	64.1 ± 0.2 (64.0 ± 0.2)	-	97 ± 2 (96 ± 3)	-	7.49 ± 0.24 (7.37 ± 0.26)
**[Dextran 40], mg mL^−1^**			***∆C*_p_, kcal mol^−^ K^−1^**		
**0**			1.60 ± 0.09		
**100**			1.59 ± 0.05		
**150**			1.58 ± 0.07		
**200**			1.58 ± 0.06		
**250** **300**			1.59 ± 0.08 1.59 ± 0.07		

**^a,b^** Have the same meaning as in Table 1. **^c^** *T*_m_ and ∆*H*_m_ in the presence of 2.0 M GdmCl. **^d^***T*_m_ and ∆*H*_m_ in the absence of 2.0 M GdmCl.

**Table 3:** Changes in stability parameters on transferring α-LA from 0 mg mL^−1^ to 300 mg mL^−1^ of crowding agents and their comparison at different pH values **^a,b^**.

pH	Dextran 40 (300 mg mL^-1^)		Dextran 70 (300 mg mL^-1^)		Ficoll 70 (300 mg mL^-1^)	
	∆*T*_m_, °C	%∆∆*G*_D_°	∆*T*_m_, °C	%∆∆*G*_D_°	∆*T*_m_, °C	%∆∆*G*_D_°
**7.0**	11.5	75.36	8.3	52.17	4.3	34.29
**6.5**	9.2	36.64	7.0	31.25	3.5	20.17
**6.0**	6.8	18.67	5.5	17.53	2.7	13.89
**5.5**	4.9	15.23	4.1	14.62	2.0	12.22

**^a^** ∆*T*_m_ = (*T*_m (crowding agent)_ − *T*_m (buffer)_). **^b^** %∆∆*G*_D_° = 100 × (Δ*G*_D_° _(crowding agent)_ − Δ*G*_D_° _(buffer)_ / Δ*G*_D_° _(buffer)_).

**Table 4:** Changes in stability parameters on transferring lysozyme from 0 mg mL^-1^ to 300 mg mL^-1^ of crowding agents and their comparison at different pH values **^a,b^**.

pH	Dextran 40 (300 mg mL^−1^)		Dextran 70 (300 mg mL^−1^)		Ficoll 70 (300 mg mL^−1^)	
	∆*T*_m_, °C	%∆∆*G*_D_°	∆*T*_m_, °C	%∆∆*G*_D_°	∆*T*_m_, °C	%∆∆*G*_D_°
**7.0**	2.1	9.84	1.7	9.29	1.0	7.34
**6.0**	2.6	11.52	2.1	9.99	1.0	6.52
**5.0**	3.3	13.46	2.2	11.32	1.1	7.28
**4.0**	5.8	17.23	2.3	12.02	1.3	6.81
**3.0**	6.1	20.70	3.0	16.23	-	-
**2.0**	6.5	33.51	4.3	25.84	-	-

**^a,b^** Have the same meaning as in Table 3.

## Supplementary Materials

The following are available online at https://www.mdpi.com/2218-273X/9/9/477/s1.

## Abbreviations

GdmCl,	guanidinium chloride;
UV,	ultra-violet;
CD,	circular dichroism;
*T*_m_,	midpoint of thermal denaturation;
Δ*H*_m_,	enthalpy change at *T*_m_;
Δ*C*_p_,	constant-pressure heat capacity change;
∆*G*_D_°,	Gibbs free energy change at 25 °C;
*K*_m_,	Michaelis constant;
*k*_cat_,	catalytic constant;
F70,	Ficoll 70;
D70,	Dextran 70;
D40,	Dextran 40.
