# Supplementary material for: Carbohydrate-Based Macromolecular Crowding-Induced Stabilization of Proteins: Towards Understanding the Significance of the Size of the Crowder
Source: Biomolecules. 2019 Sep 12;9(9):477. doi: 10.3390/biom9090477 (PMC6769620; doi:10.3390/biom9090477)
Supplement: Supplementary file 1 [file biomolecules-09-00477-s001.pdf]

SUPPLEMENTARY MATERIALS

Tables

Table S1: Thermodynamic parameters associated with the thermal unfolding of α-LA in the absence and presence of varying concentrations of ficoll 70 at different pH values[a,b].

[Ficoll 70] (mg ml^{-1})	T_m (ºC)	ΔH_m (kcal mol^{-1})	ΔG_D^o (kcal mol^{-1})
		**pH 6.5**	
**0**	50.8 ± 0.3	65 ± 3	3.52 ± 0.26
**50**	51.7 ± 0.2	65 ± 3	3.59 ± 0.28
**100**	52.3 ± 0.2	67 ± 2	3.76 ± 0.15
**150**	52.9 ± 0.1	68 ± 3	3.89 ± 0.29
**200**	53.6 ± 0.3	69 ± 3	4.04 ± 0.23
**250**	54.0 ± 0.3	70 ± 2	4.14 ± 0.16
**300**	54.3 ± 0.2	71 ± 3	4.23 ± 0.25
**350**	54.5 ± 0.3	72 ± 4	4.33 ± 0.31
		**pH 6.0**	
**0**	55.3 ± 0.2	72 ± 3	4.39 ± 0.31
**50**	55.5 ± 0.3	73 ± 3	4.51 ± 0.32
**100**	55.9 ± 0.3	74 ± 3	4.58 ± 0.30
**150**	56.5 ± 0.3	74 ± 4	4.63 ± 0.36
**200**	57.0 ± 0.2	75 ± 2	4.79 ± 0.17
**250**	57.4 ± 0.2	76 ± 3	4.89 ± 0.33
**300**	58.0 ± 0.3	77 ± 4	5.00 ± 0.37
**350**	58.2 ± 0.3	78 ± 3	5.11 ± 0.33
		**pH 5.5**	
**0**	59.7 ± 0.3<br>(59.6 ± 0.2)	76 ± 3<br>(76 ± 2)	4.99 ± 0.36<br>(4.99 ± 0.34)
**50**	59.8 ± 0.2	77 ± 2	5.13 ± 0.35
**100**	60.2 ± 0.2	78 ± 3	5.19 ± 0.46
**150**	60.6 ± 0.2	78 ± 2	5.23 ± 0.39
**200**	61.0 ± 0.3	79 ± 3	5.39 ± 0.36
**250**	61.4 ± 0.3	80 ± 3	5.50 ± 0.28
**300**	61.7 ± 0.3	81 ± 3	5.60 ± 0.38
**350**	61.9 ± 0.3<br>(61.8 ± 0.2)	82 ± 4<br>(82 ± 3)	5.72 ± 0.45<br>(5.71 ± 0.43)

[Ficoll 70], mg ml^{-1}	ΔC_p, kcal mol^{-1} K^{-1}
0	1.56 ± 0.09
50	1.55 ± 0.09
100	1.58 ± 0.06
150	1.57 ± 0.08
200	1.55 ± 0.07
250	1.56 ± 0.07
300	1.57 ± 0.06
350	1.57 ± 0.09

[a,b]Have the same meaning as in Table 1.

Table S2: Thermodynamic parameters associated with the thermal unfolding of α-LA in the absence and presence of varying concentrations of dextran 70 at different pH values[a,b].

**[Dextran 70] (mg ml^{-1})**	**T_m (°C)**	**ΔH_m (kcal mol^{-1})**	**ΔG_D^o (kcal mol^{-1})**
		**pH 6.5**	
**0**	50.8 ± 0.3	65 ± 3	3.52 ± 0.26
**50**	53.1 ± 0.3	66 ± 4	3.71 ± 0.30
**100**	54.4 ± 0.3	67 ± 3	3.87 ± 0.29
**150**	55.9 ± 0.1	68 ± 3	4.01 ± 0.31
**200**	56.9 ± 0.2	70 ± 2	4.27 ± 0.16
**250**	57.6 ± 0.2	71 ± 2	4.42 ± 0.16
**300**	57.8 ± 0.3	73 ± 3	4.62 ± 0.33
		**pH 6.0**	
**0**	55.3 ± 0.2	72 ± 3	4.39 ± 0.31
**50**	55.9 ± 0.2	73 ± 3	4.48 ± 0.32
**100**	57.4 ± 0.3	73 ± 2	4.57 ± 0.17
**150**	58.3 ± 0.3	74 ± 4	4.69 ± 0.37
**200**	59.4 ± 0.1	75 ± 2	4.88 ± 0.17
**250**	60.6 ± 0.3	76 ± 3	5.05 ± 0.35
**300**	60.8 ± 0.2	77 ± 3	5.16 ± 0.36
		**pH 5.5**	
**0**	59.7 ± 0.3<br>(59.6 ± 0.2)	76 ± 3<br>(76 ± 2)	4.99 ± 0.36<br>(4.99 ± 0.34)
**50**	59.9 ± 0.1	77 ± 3	5.07 ± 0.35
**100**	60.8 ± 0.2	78 ± 2	5.23 ± 0.40
**150**	61.6 ± 0.2	79 ± 2	5.35 ± 0.39
**200**	62.9 ± 0.3	79 ± 3	5.44 ± 0.37
**250**	63.6 ± 0.3	80 ± 3	5.60 ± 0.37
**300**	63.8 ± 0.3<br>(63.9 ± 0.2)	81 ± 2<br>(81 ± 3)	5.72 ± 0.41<br>(5.72 ± 0.41)

**[Dextran 70], mg ml^{-1}**	**ΔC_p, kcal mol^{-1} K^{-1}**
0	1.56 ± 0.09
50	1.58 ± 0.09
100	1.57 ± 0.07
150	1.58 ± 0.07
200	1.56 ± 0.08
250	1.55 ± 0.06
300	1.55 ± 0.09

[a,b]Have the same meaning as in Table 1.

Table S3: Thermodynamic parameters associated with the thermal unfolding of α-LA in the absence and presence of varying concentrations of dextran 40 at different pH values[a,b].

[Dextran 40] (mg ml^{-1})	T_m (°C)	ΔH_m (kcal mol^{-1})	ΔG_D^o (kcal mol^{-1})
	**pH 6.5**		
**0**	50.8 ± 0.3	65 ± 3	3.52 ± 0.26
**50**	53.6 ± 0.2	66 ± 2	3.75 ± 0.14
**100**	55.1 ± 0.3	68 ± 3	3.97 ± 0.24
**150**	56.6 ± 0.3	69 ± 3	4.18 ± 0.32
**200**	58.1 ± 0.2	71 ± 2	4.39 ± 0.16
**250**	59.5 ± 0.1	72 ± 2	4.57 ± 0.24
**300**	60.0 ± 0.2	74 ± 3	4.81 ± 0.35
	**pH 6.0**		
**0**	55.3 ± 0.2	72 ± 3	4.39 ± 0.31
**50**	56.1 ± 0.2	72 ± 4	4.41 ± 0.35
**100**	57.8 ± 0.3	73 ± 3	4.56 ± 0.32
**150**	58.9 ± 0.3	74 ± 2	4.77 ± 0.20
**200**	60.7 ± 0.1	75 ± 3	4.89 ± 0.36
**250**	61.9 ± 0.2	76 ± 3	5.07 ± 0.37
**300**	62.1 ± 0.2	77 ± 3	5.21 ± 0.37
	**pH 5.5**		
**0**	59.7 ± 0.3<br>(59.6 ± 0.2)	76 ± 3<br>(76 ± 2)	4.99 ± 0.36<br>(4.99 ± 0.34)
**50**	60.1 ± 0.1	76 ± 3	5.00 ± 0.35
**100**	61.0 ± 0.2	77 ± 3	5.10 ± 0.36
**150**	61.9 ± 0.3	78 ± 4	5.31 ± 0.42
**200**	63.2 ± 0.1	79 ± 2	5.40 ± 0.19
**250**	64.3 ± 0.3	80 ± 3	5.60 ± 0.39
**300**	64.6 ± 0.3<br>(64.5 ± 0.2)	81 ± 3<br>(81 ± 2)	5.75 ± 0.38<br>(5.74 ± 0.37)

**[Dextran 40], mg ml^{-1}**	**ΔC_p, kcal mol^{-1} K^{-1}**
0	1.56 ± 0.09
50	1.57 ± 0.09
100	1.59 ± 0.07
150	1.55 ± 0.08
200	1.58 ± 0.08
250	1.56 ± 0.09
300	1.55 ± 0.09

[a,b]Have the same meaning as in Table 1.

Table S4: Thermodynamic parameters associated with the thermal unfolding of lysozyme in the absence and presence of varying concentrations of dextran 40 at different pH values[a,b].

[Dextran 40] (mg ml^{-1})	$T_{m\,(obs.)}$[c] (ºC)	$T_{m\,(corr.)}$[d] (ºC)	$\Delta H_{m\,(obs.)}$[c] (kcal mol^{-1})	$\Delta H_{m\,(corr.)}$[d] (kcal mol^{-1})	ΔG_D^o (kcal mol^{-1})
			**pH 6.0**		
**0**	57.9 ± 0.3	83.3 ± 0.3	86 ± 3	125 ± 3	12.41 ± 0.30
**100**	58.5 ± 0.2	83.9 ± 0.2	87 ± 3	126 ± 3	12.59 ± 0.44
**150**	59.2 ± 0.3	84.6 ± 0.3	88 ± 2	127 ± 2	12.83 ± 0.21
**200**	59.5 ± 0.3	84.9 ± 0.3	91 ± 4	130 ± 4	13.34 ± 0.59
**250**	60.3 ± 0.2	85.7 ± 0.2	93 ± 3	132 ± 3	13.66 ± 0.47
**300**	60.5 ± 0.3	85.9 ± 0.3	94 ± 2	133 ± 2	13.84 ± 0.34
			**pH 5.0**		
**0**	55.8 ± 0.3	80.2 ± 0.3	83 ± 3	121 ± 3	11.66 ± 0.24
**100**	57.6 ± 0.2	82.0 ± 0.2	84 ± 3	122 ± 3	11.88 ± 0.43
**150**	57.7 ± 0.3	82.1 ± 0.3	86 ± 2	124 ± 2	12.25 ± 0.20
**200**	58.1 ± 0.2	82.5 ± 0.2	88 ± 3	126 ± 3	12.59 ± 0.37
**250**	58.9 ± 0.3	83.3 ± 0.3	90 ± 3	128 ± 3	12.90 ± 0.44
**300**	59.1 ± 0.3	83.5 ± 0.3	92 ± 2	130 ± 2	13.23 ± 0.34
			**pH 4.0**		
**0**	52.2 ± 0.3	76.8 ± 0.3	79 ± 3	115 ± 3	10.56 ± 0.30
**100**	53.2 ± 0.3	77.8 ± 0.3	80 ± 3	116 ± 3	10.79 ± 0.40
**150**	53.5 ± 0.3	78.1 ± 0.3	81 ± 3	117 ± 3	10.99 ± 0.39
**200**	55.8 ± 0.2	80.4 ± 0.2	84 ± 2	120 ± 2	11.55 ± 0.27
**250**	56.9 ± 0.2	81.5 ± 0.2	86 ± 3	122 ± 3	11.86 ± 0.42
**300**	58.0 ± 0.1	82.6 ± 0.1	89 ± 3	125 ± 3	12.38 ± 0.43
			**pH 3.0**		
**0**	69.7 ± 0.2	-	103 ± 2	-	8.50 ± 0.23
**100**	70.8 ± 0.3	-	104 ± 2	-	8.77 ± 0.28
**150**	71.4 ± 0.3	-	106 ± 2	-	9.10 ± 0.26
**200**	73.2 ± 0.2	-	108 ± 3	-	9.46 ± 0.38
**250**	74.6 ± 0.2	-	111 ± 2	-	9.91 ± 0.28
**300**	75.8 ± 0.3	-	113 ± 2	-	10.26 ± 0.30

[Dextran 40], mg ml^{-1}	ΔC_p, kcal mol^{-1} K^{-1}
**0**	1.60 ± 0.09
**100**	1.59 ± 0.05
**150**	1.58 ± 0.07
**200**	1.58 ± 0.06
**250**	1.59 ± 0.08
**300**	1.59 ± 0.07

[a,b]Have the same meaning as in Table 1.
[c,d]Have the same meaning as in Table 2.

Table S5: Kinetic parameters of lysozyme in the absence and presence of different concentrations of dextran 40 at pH 7.0 and 25 °C[a].

**[Dextran 40] (mg ml^{-1})**	$\boldsymbol{K_m}$ **(mg l^{-1})**	$\boldsymbol{k_{cat}}$ **x 10^{-5} (mg s^{-1} M^{-1})**
**0**	83.75 ± 5.17	9.64 ± 0.09
**100**	77.45 ± 6.52	8.27 ± 0.09
**150**	71.97 ± 3.44	8.09 ± 0.06
**200**	67.13 ± 3.81	7.53 ± 0.09
**250**	63.24 ± 5.22	7.35 ± 0.06
**300**	56.05 ± 4.07	7.05 ± 0.05

[a]Have the same meaning as in Table 1.

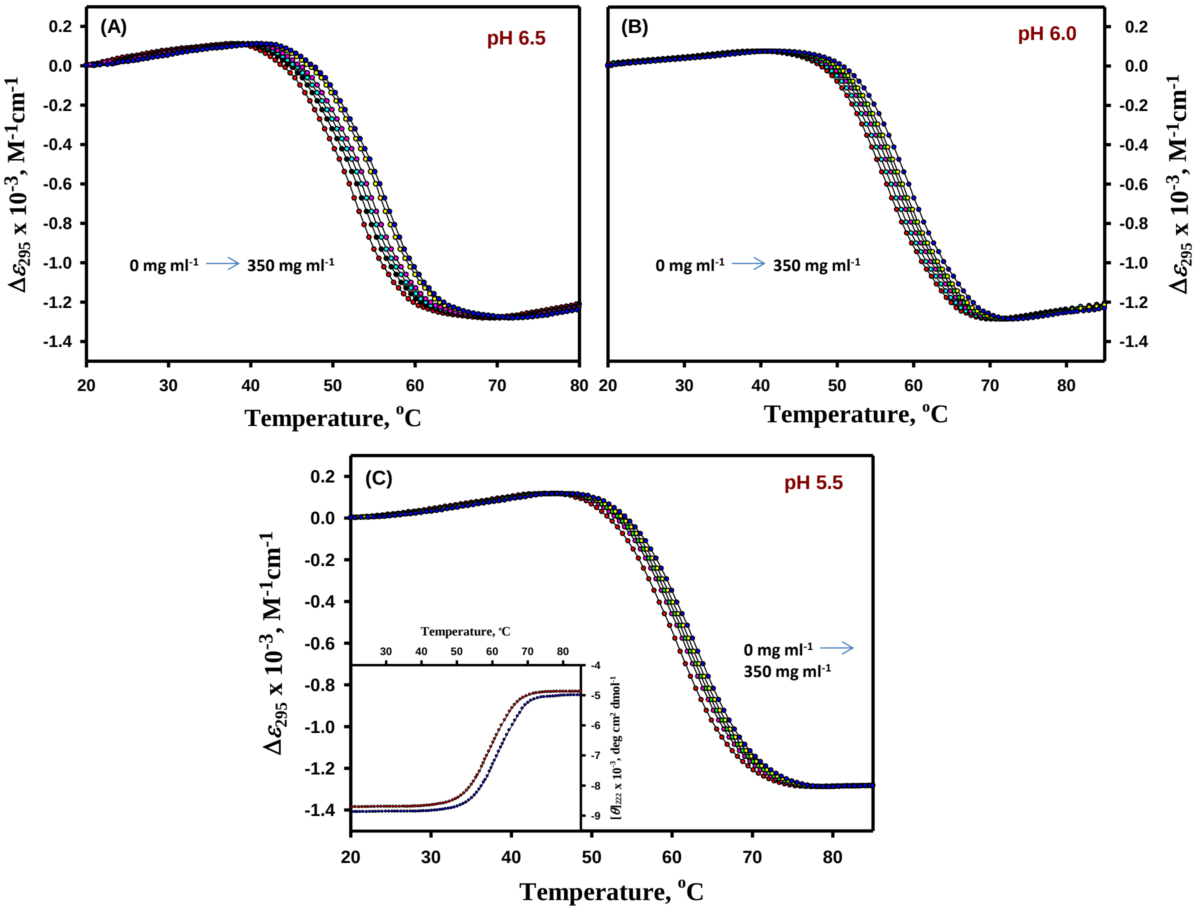

Figure S1. Thermal denaturation profiles of α-LA in the absence and presence of different concentrations of ficoll 70 at different pH values (panels A-C). Different concentrations of the crowding agent are shown by different colours: 0 mg ml^{-1} (red), 50 mg ml^{-1} (black), 100 mg ml^{-1} (cyan), 150 mg ml^{-1} (pink), 200 mg ml^{-1} (green), 250 mg ml^{-1} (yellow) and 350 mg ml^{-1} (blue). For the sake of clarity curves at all concentrations are not shown. Inset in panel C represents thermal denaturation profiles of α-LA measured by $[\theta]_{222}$ in the absence (red circle) and presence of the highest concentration of ficoll 70 (blue circle).

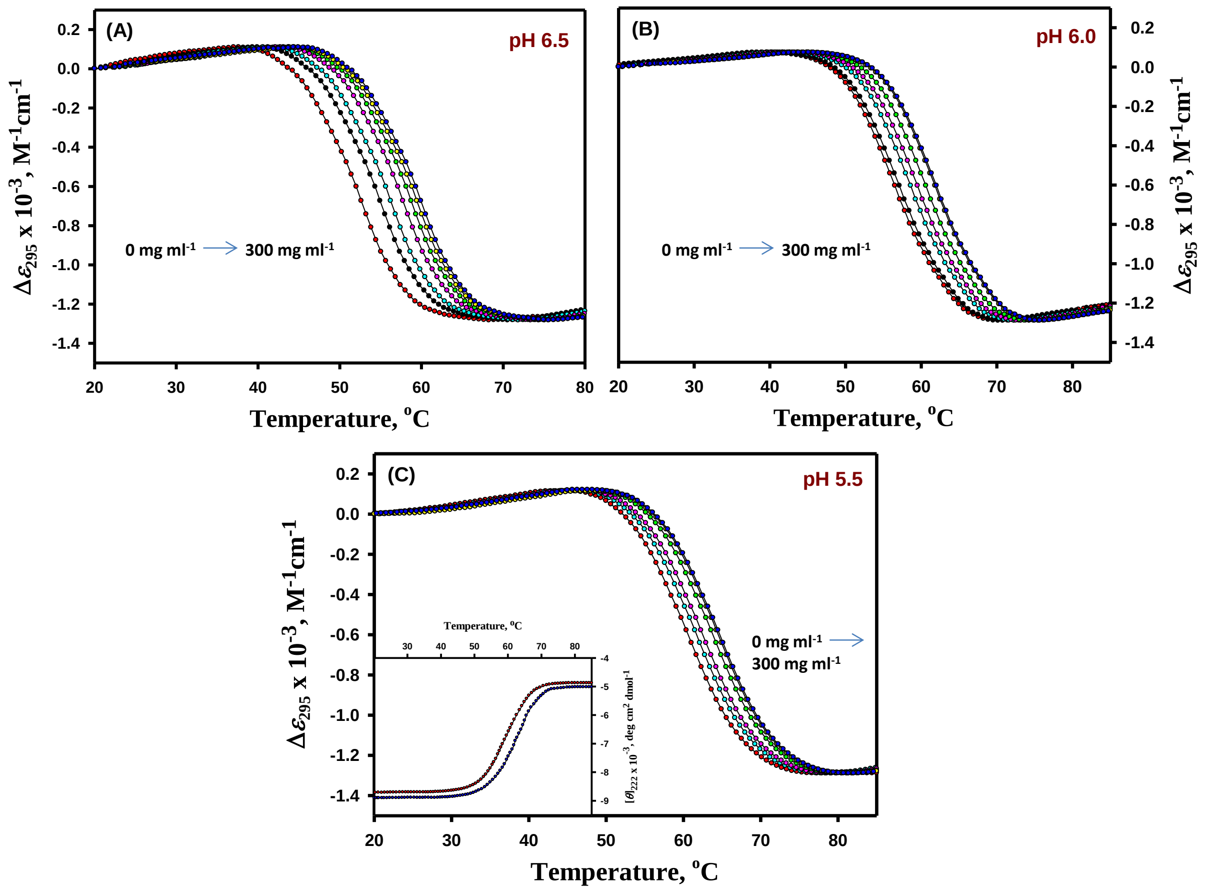

Figure S2. Thermal denaturation profiles of α-LA in the absence and presence of different concentrations of dextran 70 at different pH values (panels A-C). Different concentrations of the crowding agent are shown by different colours: 0 mg ml^{-1} (red), 50 mg ml^{-1} (black), 100 mg ml^{-1} (cyan), 150 mg ml^{-1} (pink), 200 mg ml^{-1} (green), 250 mg ml^{-1} (yellow) and 300 mg ml^{-1} (blue). For the sake of clarity curves at all concentrations are not shown. Inset in panel C represents thermal denaturation profiles of α-LA measured by $[\theta]_{222}$ in the absence (red circle) and presence of highest concentration of dextran 70 (blue circle).

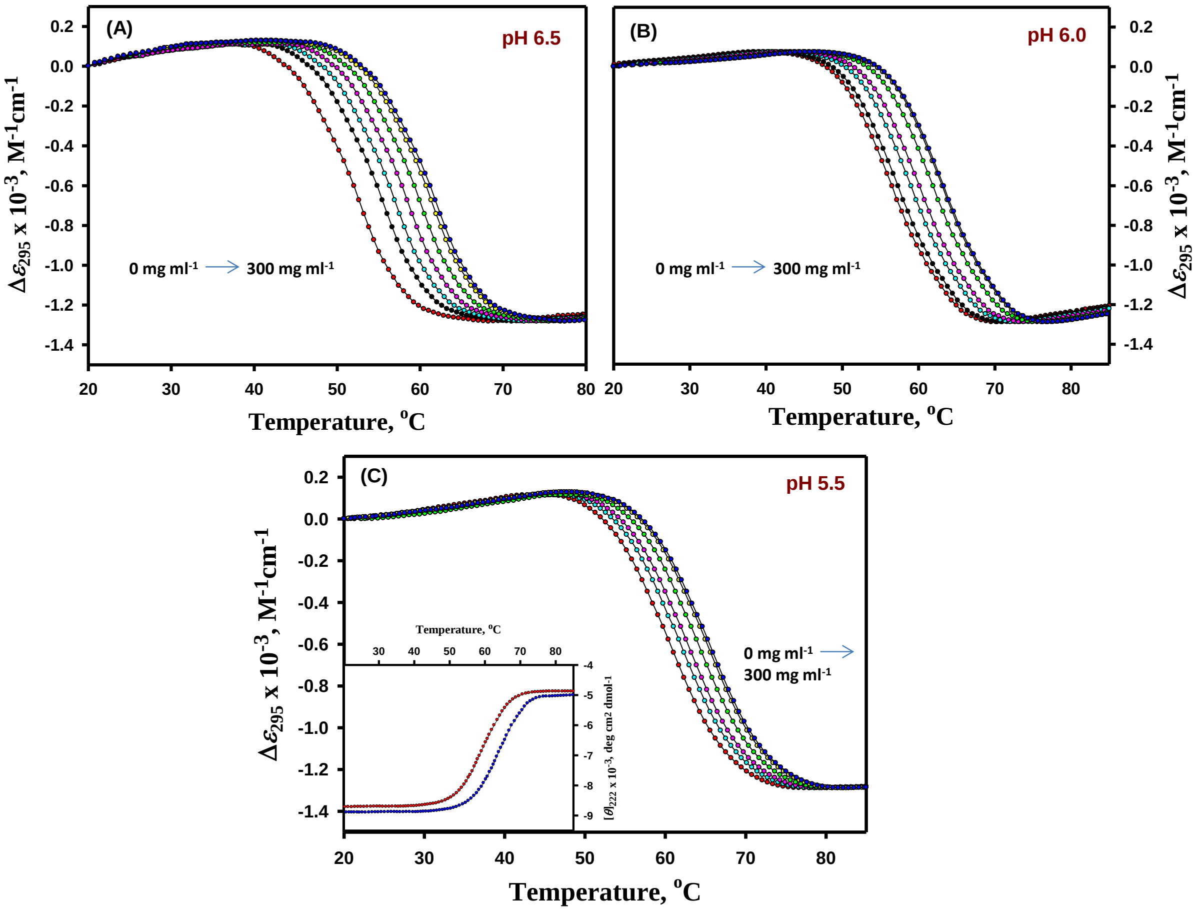

Figure S3. Thermal denaturation profiles of α-LA in the absence and presence of different concentrations of dextran 40 at different pH values (panels A-C). Different concentrations of the crowding agents are shown by different colours: 0 mg ml^{-1} (red), 50 mg ml^{-1} (black), 100 mg ml^{-1} (cyan), 150 mg ml^{-1} (pink), 200 mg ml^{-1} (green), 250 mg ml^{-1} (yellow) and 300 mg ml^{-1} (blue). For the sake of clarity curves at all concentrations are not shown. Inset in panel C represents thermal denaturation profiles of α-LA measured by $[\theta]_{222}$ in the absence (red circle) and presence of highest concentration of dextran 40 (blue circle).

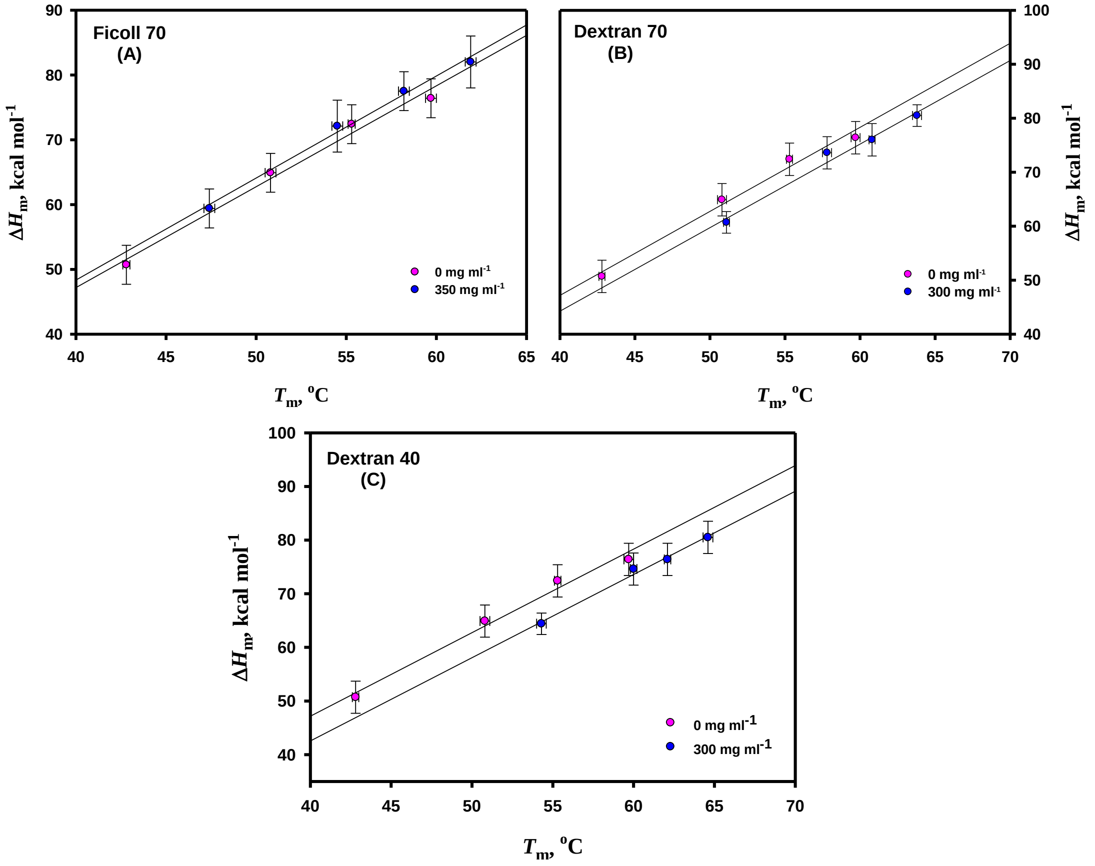

Figure S4. Plots of ΔH_m versus T_m of α-LA in the absence (pink) and presence of highest concentration (blue) of all the crowders at different pH values. ΔC_p is estimated from the slope of each plot.

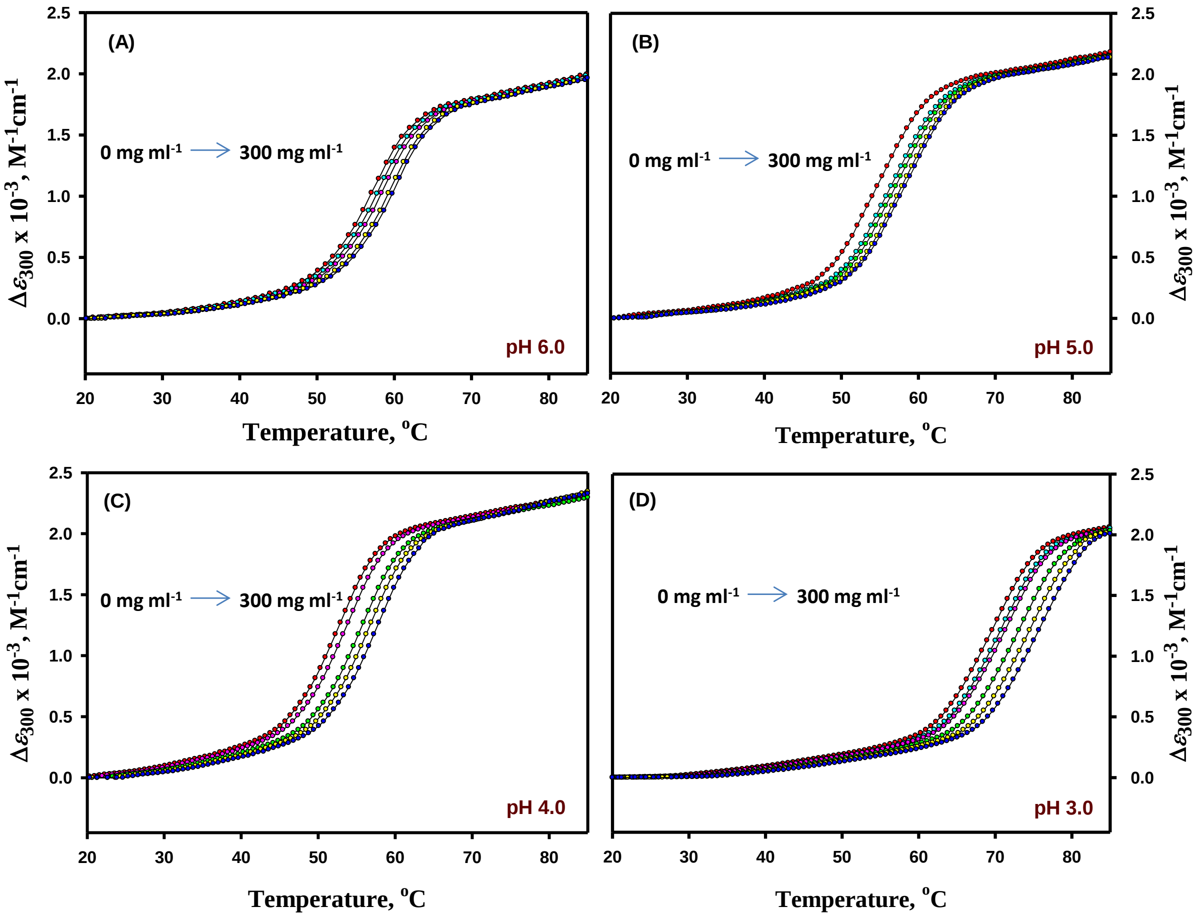

Figure S5. Thermal denaturation profiles of lysozyme in the absence and presence of different concentrations of dextran 40 at different pH values (panels A-D). Different concentrations of dextran 40 are shown by different colours: 0 mg ml^{-1} (red), 100 mg ml^{-1} (cyan), 150 mg ml^{-1} (pink), 200 mg ml^{-1} (green), 250 mg ml^{-1} (yellow) and 300 mg ml^{-1} (blue). For the sake of clarity curves at all concentrations are not shown.

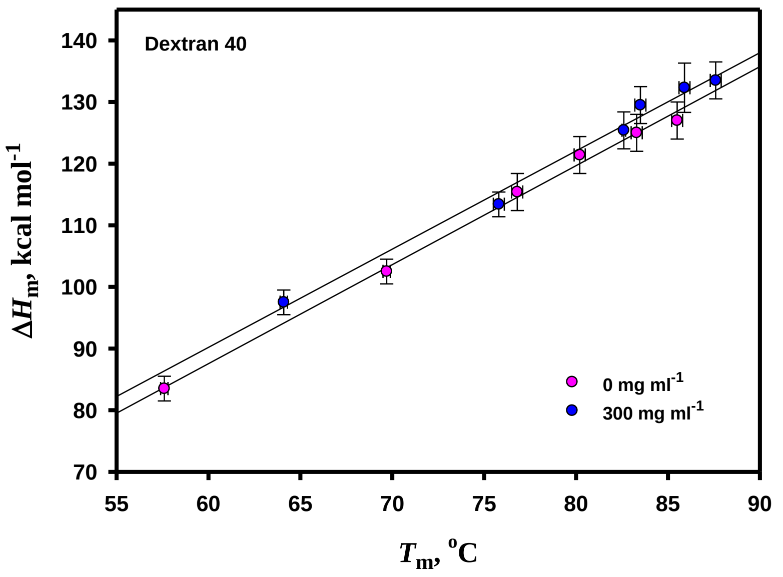

Figure S6. Plots of ΔH_m versus T_m of lysozyme in the absence (pink) and presence of the highest concentration (blue) of dextran 40 at different pH values. ΔC_p is estimated from the slope of each plot.

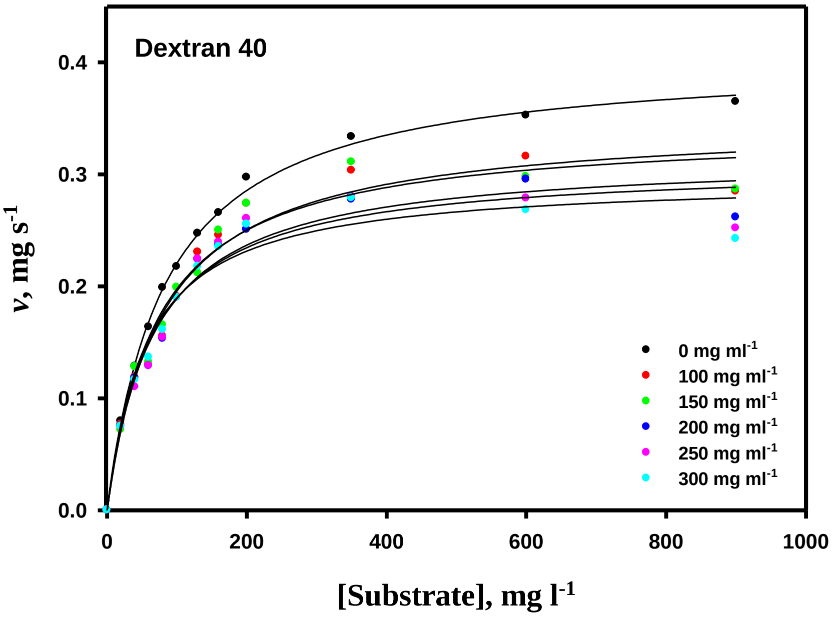

Figure S7. Plots of Initial velocity (*v*) versus [substrate], the substrate concentration for lysozyme in the absence and presence of different concentrations of dextran 40 at pH 7.0 and 25 °C.
